# Immunomodulatory effects of *Stachytarpheta cayennensis* leaf extract and its synergistic effect with artesunate

**DOI:** 10.1186/1472-6882-14-376

**Published:** 2014-10-05

**Authors:** Theophine C Okoye, Peter A Akah, Adaobi C Ezike, Philip F Uzor, Uchenna E Odoh, Sebastian O Igboeme, Uchechi B Onwuka, Sunday N Okafor

**Affiliations:** Department of Pharmacology & Toxicology, Faculty of Pharmaceutical Sciences, University of Nigeria, Nsukka, Enugu State 410001 Nigeria; Department of Pharmaceutical & Medicinal Chemistry, Faculty of Pharmaceutical Sciences, University of Nigeria, Nsukka, Enugu State 410001 Nigeria; Department of Pharmacognosy & Environmental Medicines, Faculty of Pharmaceutical Sciences, University of Nigeria, Nsukka, Enugu State 410001 Nigeria; Department of Clinical Pharmacy & Pharmacy management, Faculty of Pharmaceutical Sciences, University of Nigeria, Nsukka, Enugu State 410001 Nigeria

**Keywords:** Cellular mediated immunity, Delayed type hypersensitivity, Immunomodulatory action, *Stachytarpheta cayennensis*, Artesunate

## Abstract

**Background:**

The leaves of *Stachytarpheta cayennensis* C. Rich. (Verbenaceae) have been reported to possess potent anti-inflammatory and anti-malarial activities. Due to close association between inflammatory and immune responses, we evaluated the immunomodulatory activity of leaves extract of *S. cayennensis*. The combined effects of the leaves extract and artesunate, a standard antimalarial agent with immunomodulatory effects, were also evaluated.

**Methods:**

The immunomodulatory activity of the methanol extract of the leaves of *S. cayennensis* (MESC) was evaluated in mice using the Delayed-Type hypersensitivity response (DTHR), primary and secondary humoral immune responses and the *in vivo* leucocyte mobilization tests. The immunomodulatory effect of artesunate and the combined effects of MESC and artesunate were evaluated using the phagocytic activity of polymorphonuclear neutrophils. Acute toxicity and lethality test in addition to the preliminary phytochemical studies of MESC were also performed.

**Results:**

The MESC exhibited 64.21% inhibition of DTHR at 500 mg/kg dose and evoked 139.64% of phagocytic stimulation at 100 μg/ml dose. Also MESC significantly (p < 0.05) showed dose related stimulation of humoral immunity and a highest percentage leucocyte mobilization of 10.15% at 250 mg/kg dose. Artesunate offered a non-significant (p > 0.05) percentage phagocytic stimulation (PPS) while the combined effect of artesunate and MESC exhibited a significant (p < 0.05) dose dependent PPS with highest PPS of 393.77% at 100 μg/ml. The LD_50_ of the MESC was estimated to be greater than 5000 mg/kg since there were no lethality and signs of acute intoxication after 48 h observation. Preliminary phytochemical analysis revealed the presence of carbohydrates, glycosides, flavonoids, saponins, alkaloids, terpenoids and steroids.

**Conclusions:**

The results of this study indicated that MESC possesses immunostimulatory action with significant synergistic effects with artesunate, and can therefore, offer immune boosting activities in disorders of immune suppression.

## Background

The scourge of acquired immune deficiency syndrome (AIDS) and other related immune deficiency diseases such as ebola virus disease and tuberculosis, especially in the developing countries, are established health concerns. In most cases, the suppression of immune system not only attracts opportunistic infections but also adversely affects the quality of life of patients. These, together with the attendant co-morbidity of immune disorders have recently stimulated research interest in the area of immune boosting potentials of medicinal plants.

The human immune system consists of two categories of defense mechanisms; the innate (non-specific) and the adaptive (specific) systems. These two mechanisms could be modified by substances, such as drugs and plant constituents, to either enhance or suppress their ability to resist invasion by pathogens [1, 2]. The concern about the safety and adverse effects of the conventional immunomodulatory synthetic drugs has led to a growing interest in identifying and characterizing natural compounds with immunomodulatory effects possessing low profile toxicity [3, 4].

Medicinal plants have been reported to possess numerous secondary metabolites with potent immunomodulatory effects [3, 5, 6]. Most of these plants or plant parts are being used in folkloric medicine by rural dwellers to boost their immunity against infections. Studies have shown that many plants with proven anti-inflammatory effect also possess immunomodulatory effects based on the relationship between physiological mechanisms of inflammation and immune responses [7–9]. Some medicinal plants with established immunomodulatory effects are *Panax ginseng*, *Viscum album*, *Tinospora cordifolia*, *Boerhaavia diffusa*, *Withania somnifera*, *Ocimum sanctum* and *Curculigo orchioides* [10–15]. In addition, the immunomodulatory effects of artesunate, an artemisinin derivative isolated from the plant, *Artemisia annua* and related agents have also been documented [16, 17].

The leaves of *Stachytarpheta cayennensis* C. Rich (Verbenaceae) have been documented to possess potent analgesic, antimalarial and antiinflammatory effects [18–20]. *S. cayennensis,* commonly known as “Gervâo” is a weedy annual (and sometimes perennial) herbaceous plant that grows mainly in the tropical rainforests, indigenous to most parts of tropical America, Mexico, Haiti, West Indies and South America [21]. The plant is used traditionally as anti-allergic, bronchodilatory, digestion-stimulating, antacid and antidiarrhea [21], pain-relieving, antispasmodic and antiinflammatory [22, 20], liver protective and detoxification [23], cellular protective, antioxidant and antimicrobial [22, 24], anticonvulsant and sedative [25] agents.

The present study investigated the immunomodulatory potentials of methanol extract of *S. cayennensis* (MESC) leaves on cellular and humoral immune responses and assessed its synergistic effect with artesunate, a standard antimalarial agent with immunomodulatory potentials.

## Methods

### Collection and preparation of plant material

Fresh leaves of *S. cayennensis* were collected in the month of July, from Nsukka, Enugu State, Nigeria. The plant was identified and authenticated by Mr. Alfred Ozioko, a taxonomist of the International Center for Ethnomedicine and Drug Development (InterCEDD), Aku Road, Nsukka, Enugu State, Nigeria. The voucher specimen was deposited at the herbarium of the InterCEDD, Nsukka, Nigeria. The leaves were cleaned, cut into small pieces, dried and pulverized to coarse powder using a manual blender.

### Extraction of plant material

The powdered dried leaves (700 g) were extracted with methanol using cold maceration for 48 h. The entire bulk was filtered and concentrated using rotary evaporator under reduced pressure to obtain the methanol extract of the leaves of *S. cayennensis* (MESC; 13.6% w/w) which was subjected to various tests. The MESC before administration was prepared by dissolving it in distilled water and diluting to appropriate concentration in the course of each experiment.

### Materials

Pure artesunate sample (Emzor Pharma Nig. Ltd, Lagos) and methanol extract of the leaves of *S. cayennensis* (MESC).

### Animals

Adult Swiss albino mice (22-30 g) and rat (200 g) of both sexes were obtained from the Animal House Facility of the Department of Pharmacology and Toxicology, University of Nigeria, Nsukka and used for this study. The animals were housed under standard conditions (25 ± 2°C) with free access to standard pellets (Guinea Feed Nigeria, Ltd.) and water. A healthy male sheep (for antigen used in Delayed Type Hypersensitivity Reaction test) was taken from the herd in the experimental animal facility of the Faculty of Veterinary Medicine, University of Nigeria, Nsukka. The sheep had free access to normal pasture in the farm. The animals (mice and rat) were transferred to the research area and were allowed a 14 day acclimatization period before the experiments. All animal experiments were conducted in compliance with the National Institute of Health Guide for Care and Use of Laboratory Animals (Pub No. 85-23, revised 1985) and in accordance with the University of Nigeria Ethics Committee on the use of laboratory animals, registered by the National Health Research Ethics Committee (NHREC) of Nigeria, with the number; NHREC/05/01/2008B. The protocols employed were submitted to and approved by the University Ethics Committee.

### Antigen

Fresh sheep blood (10 ml) was aseptically taken from the jugular vein of a healthy male sheep and transferred to heparinized tube. The blood samples were washed thrice in about 5-10 ml of pyrogen-free sterile normal saline by centrifugation at 3000 rpm for 10 min on each occasion. The washed SRBCs was adjusted to a concentration of 1 × 10^9^ cells/ml with sterile normal saline and used for immunization and challenge.

### Micro-organism

Clinical isolates of *Candida albicans* obtained from the Microbiology Unit of the Department of Pharmaceutics and Pharmaceutical Microbiology, University of Nigeria, Nsukka. The *C. albicans* had been isolated from a high vaginal swab in the Medical Laboratory of a local hospital at Nsukka, Nigeria.

### Phytochemical analysis

Qualitative phytochemical analysis was performed on MESC using standard procedures outlined by Harborne [26] and Trease and Evans [27]. Briefly, frothing test for saponins, Salkowski test for terpenoids, Liebermann-Burchard tests for steroids, ferric chloride test for tannins, Keller-Killiani test for cardiac glycosides, Dragendorff’s and Mayer’s test for alkaloids, Fehling’s test for reducing sugars, xanthoproteic test for proteins, iodine test for carbohydrates or starch and ammonia test for detection of flavonoids were performed for qualitative identification of the phytoconstituents present [28]. All reagents used for the phytochemical analysis were freshly prepared.

### Acute toxicity study

The acute lethal dose (LD_50_) of MESC was ascertained by the method described by Lorke [29]. Briefly, the study was performed in two phases. In the first phase, 9 mice were divided into 3 groups of 3 mice per group, and treated with the MESC at the doses of 10, 100 and 1000 mg/kg (p.o.) respectively. The animals were observed for 24 h for signs of toxicity. In the second phase, four mice were used. Three were treated separately with MESC doses of 1600, 2900 and 5000 mg/kg respectively, while the fourth (the control) received 10 ml/kg of distilled water. The animals were observed for 24 h period.

### Preparation of *Candida albicans*suspension

*Candida albicans* culture was incubated in Sabouraud dextrose broth overnight and centrifuged to form a cell button at the bottom of the test tube. The supernatant was discarded and the cell button was washed 3-4 times with sterile phosphate buffer saline (PBS) and centrifuged. The washed cell button was re-suspended in a mixture of PBS and rat serum in the proportion of 4:1. The count of *C. albicans* was adjusted to 1 × 10^8^ cells/ml using the 0.5 McFarland standard.

### Preparation of the slide and evaluation of phagocytosis

About 0.2 ml of rat blood was smeared on sterile glass slide and incubated at 37°C for 20 min for clotting to occur. The slide was thereafter drained slowly with sterile normal saline in order not to wash off the adhered neutrophils or polymorphonuclear (PMN) leucoccytes. The slide consisting of neutrophils was flooded with the MESC, artesunate and combinations of the MESC and artesunate at different concentrations (25, 50 and 100 μg/ml), and were incubated at 37°C for 20 min. The PMNs were covered with *C. albicans* suspension and incubated at 37°C for 1 h. The slide was drained, fixed with methanol and stained with Giemsa stain and observed (100 X) under oil immersion and phagocytosis evaluated by the method described by Ganachari *et al*. [30]. The number of *C. albicans* cells phagocytosed by PMNs on the slide was determined microscopically for granulocytes using morphological criteria. This number was regarded as Phagocytic Index (PI) and was compared with the PI of the control treatment. Immunostimulation was calculated using the following equation:


### Determination of Delayed Type Hypersensitivity Response (DTHR)

Twenty mice were randomly divided into four groups consisting of five animals per group. Delayed type hypersensitivity was induced in mice using Sheep Red Blood Cells (SRBCs). The MESC, suspended in distilled water, was administered by oral gavage to the animals. Groups 1, 2 and 3 received 100, 250 and 500 mg/kg of the extract respectively while group 4 received levamisole (control). On day 0, one hour after extract administration, the rats were sensitized by injecting 0.02 ml of 10^9^ cells/ml of SRBCs subcutaneously, into the right hind foot paw. The animals were challenged on day 5 by subcutaneous injection of the same amount of antigen (SRBCs) into the left hind foot paw. The animals were treated with the extract 3 days prior to sensitization and continued till the challenge. The oedema produced by antigenic challenge in the left hind paw was measured as the difference in the paw thickness before and 24 h after the challenge. The paw thickness was measured with a pocket-sized screw gauge [8, 2, 31].

### Determination of humoral immune response

Twenty mice were randomly selected and divided into four groups of five animals in each group. The MESC, suspended in distilled water, was administered by oral gavage to the animals. Groups A, B and C received 100, 250 and 500 mg/kg body weight of MESC respectively, while group D (control) received distilled water. The method of Nelson and Mildenhall [32] was used to determine the effect of MESC on the antibody level of the animals resulting from sensitization with SRBCs. Briefly, on day zero, 0.1 ml of the 10^9^ cell/ml SRBCs was given by intra-peritoneal injection (i.p.) to all the groups for immunization. The animals were challenged on day 5 by similar i.p. injection of the same amount of 10^9^ cell/ml SRBCs. The MESC was administered 3 days prior to sensitization and continued daily for 5 days after the challenge [2]. Primary antibody titre was determined on day 5 (before the challenge) and secondary titre on day 10. Blood samples were obtained by retro-orbital puncture into test tubes and allowed to clot. A 25 μl serum for each sample was obtained after centrifugation and serially diluted two-fold in 96-U well microtitre plates using pyrogen-free serial normal saline. The last well on each row contained sterile normal saline as control. The diluted sera were challenged with 25 μl of 1% v/v SRBCs in the plates and then incubated at 37°C for 1 h. The highest dilution giving rise to visible heamagglutination was taken as antibody titre. Antibody titres were expressed in graded manner, the minimum dilution (1/2) being ranked as 1 (calculated as log_2_ of the dilution factor).

### *In vivo*leucocyte mobilization test

The method of Ribeiro *et al*. [33] was used in the test for the effect of MESC on the *in vivo* leucocyte mobilization induced by inflammatory stimulus. Mice were divided into four groups of five animals each. Groups I – III received MESC (100, 250 and 500 mg/kg, respectively) while group IV received the vehicle. One hour after the administration of the extract, each mice in the groups received injection (i.p.) of 0.5 ml of 3% w/v agar suspension in normal saline. Four hours later, the mice were sacrificed and the peritoneum washed with 5 ml of a 5% solution of EDTA in Phosphate Buffered Saline (PBS). Total and differential leucocyte counts (TLC and DLC) were performed on the perfusates while the percentage leucocyte mobilization (PLM) was calculated using the formula; PLM (%) = TLC (Test) – TLC (Control)/TLC (Control) × 100.

### Statistical analysis

The results from the experiment were analyzed using one way Analysis of variance (ANOVA; Dunnett post hoc test) and expressed as the mean values for each group ± SEM. The statistical significance between the test and the control groups were considered at p < 0.05.

## Results

### Preliminary phytochemical analysis

Results of preliminary phytochemical analysis of the MESC showed the presence of carbohydrates, glycosides, flavonoids, saponins, alkaloids, terpenoids and steroids (Table 1).

**Table 1:** **Phytochemical constituents of the leaf extract of** ***S. cayennensis***

Constituent	MESC
Carbohydrates	+
Reducing sugar	+
Glycosides	+
Flavonoids	+
Saponins	+
Alkaloids	+
Terpenoids	+
Tannins	-
Resins	-
Steroids	+

+ = present; - = absent.

### Acute toxicity and lethality test

The MESC exhibited an estimated LD_50_ greater than 5000 mg/kg (per oral) and did not cause any lethality and signs of acute intoxication after 48 h observation period. This was in line with other documented work on the leaves of *S. cayennensis* [24, 23].

### Effect on phagocytic activity of polymorphonuclear leucocytes

**Figure 1:**
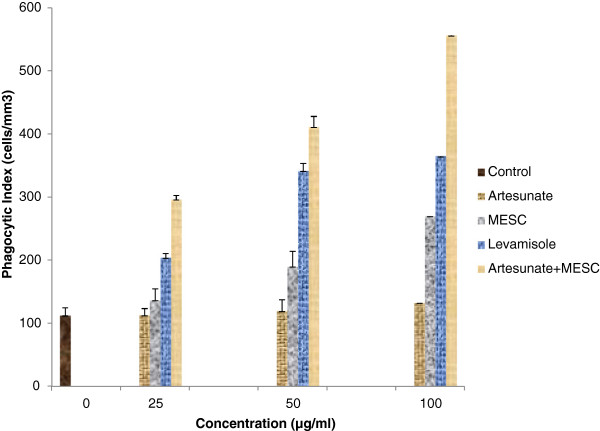
**Phagocytic indexes (PI) of methanol extract of** ***Stachytarpheta cayennensis*** **(MESC), levamisole, artesunate and the combined effects of MESC and artesunate.** PI showed the degree of phagocytic activities of polymorphonuclear leucocytes.

**Figure 2:**
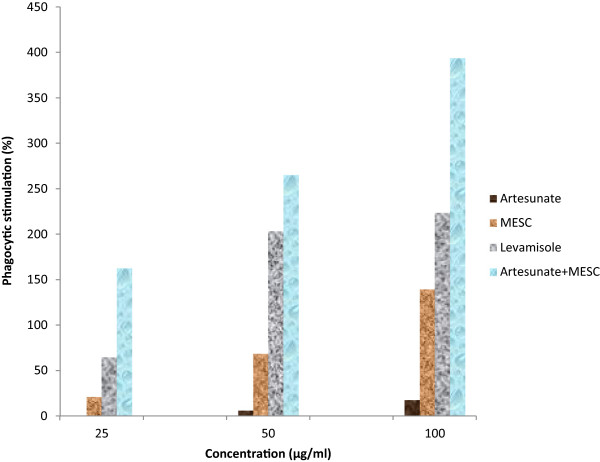
**Percentage of phagocytic stimulation of the** ***Stachytarpheta cayennensis*** **extract (MESC), levamisole, artesunate and the combined effects of MESC and artesunate.**

Artesunate at the doses tested exhibited non-significant (p > 0.05) phagocytic stimulation compared to the control (Figure 1). MESC also at doses tested exhibited no significant (p > 0.05) phagocytic stimulation except the 100 μg/ml dose which exhibited significant (p < 0.05) phagocytic stimulation compared to the control. Moreover, the combined doses of the MESC and artesunate exhibited significant (p < 0.05) phagocytic stimulation (Figure 1). At 100 μg/ml concentration, artesunate, MESC, levamisole and artesunate plus MESC showed percentage phagocytic stimulation of 17.75, 139.64, 223.66 and 393.77% respectively (Figure 2). In addition the combined doses of the MESC and artesunate exhibited the highest phagocytic index (PI) and percentage phagocytic stimulation compared to the effects of the separate doses, the control and the levamisole, a standard agent (Figures 1 & 2).

### Determination of Delayed Type Hypersensitivity Response (DTHR)

The MESC at the doses tested showed significant (p < 0.05) inhibition of delayed type hypersensitivity response. The extract, at 500 mg/kg dose, exhibited highest percentage inhibition of the DTHR of 64.21% greater than that of levamisole which was 58.05% (Table 2).

**Table 2:** **The effect MESC on delayed type hypersensitivity reaction**

Treatment	Dose (mg/kg)	Paw thickness (mm)	Inhibition of DTHR (%)
MESC	100	0.840 ± 0.07*	55.79
MESC	250	0.770 ± 0.03*	59.47
MESC	500	0.680 ± 0.06*	64.21
Levamisole	2.5	0.797 ± 0.22*	58.05
Control	-	1.900 ± 0.07	-

Values are expressed as the mean ± SEM using ANOVA, Dunnet as Post hoc.

*p < 0.05 compared with the control; n = 5.

### Determination of humoral immune response

The MESC caused dose dependent statistically significant (p < 0.05) elevation in the secondary antibody titres at the doses treated compared with the control. Moreover, MESC at all doses tested exhibited increased secondary mean titre values from the primary means titre values (Table 3).

**Table 3:** **The effect of MESC on primary and secondary humoral immune response**

Treatment	Dose (mg/kg)	Primary (mean titre)	Secondary (mean titre)
MESC	100	2.83 ± 0.17	3.00 ± 0.00
MESC	250	3.38 ± 0.43	3.52 ± 0.87*
MESC	500	3.75 ± 0.25	4.25 ± 0.87*
Control	-	3.75 ± 0.25	2.30 ± 0.45

Values are expressed as the mean ± SEM using ANOVA, Dunnet as Post hoc.

*p < 0.05 compared with the control; n = 5.

### *In vivo*leucocytes mobilization test

The result of the *in vivo* leucocyte mobilization showed that MESC produced a dose-dependent increase in the peritoneal percentage leucocyte mobilization up to the 250 mg/kg dose as well as that of the neutrophil differential analysis. However, MESC showed a non-dose-dependent mobilization of the lymphocyte differential analysis while the 250 mg/kg dose exhibited highest percentage leucocyte mobilization of 10.15% (Table 4).

**Table 4:** **The effect of MESC on** ***in-vivo*** **Leucocytes mobilization**

Treatment	Dose (mg/kg)	TLC (cell mm ^-3^)	Leucocytes mobilization (%)	Differentials	
				Neutrophil	Lymphocytes
MESC	100	46.50 ± 4.99	-5.58	0.75 ± 0.16	44.80 ± 4.69
MESC	250	54.25 ± 18.20	10.15	4.49 ± 1.84	29.67 ± 11.97
MESC	500	50.25 ± 6.57	2.03	3.79 ± 1.38	46.54 ± 5.56
Control	-	49.25 ± 6.10	-	4.15 ± 0.78	45.11 ± 6.67

Values are expressed as the mean ± SEM using ANOVA, Dunnet as Post hoc, n = 5.

## Discussion

Findings from this study showed that MESC exhibited potent immunomodulatory effect on both humoral mediated and cell mediated immune responses. On humoral mediated immune response, MESC enhanced antibody synthesis whereas it stimulated phagocytic activity of polymorphonuclear leucocytes (PMNs) and suppressed the delayed type hypersensitivity reaction induced by SRBCs which are all cell-mediated events. It also enhanced leukocyte migration under inflammatory stimulus.

The increase in antibody titre evoked by MESC clearly indicates stimulation of the humoral immunity. Humoral-mediated immunity involves the production specific antibodies (immunoglobulins) by bursa equivalent lymphocytes or plasma cells following sensitization to specific antigen [34]. Humoral immunity or antibody-mediated immunity is part of the body’s adaptive immune response produced by the B-cell lymphocytes responsible for antibody production. It is activated by the alpha globulin antibodies on contact with antigenic substances such as proteins or polysaccharides foreign to the body. By stimulating antibody production, MESC may augment the body’s immunity and enhance its capacity to contain infections of both bacterial and viral origins, which are organisms responsible for body’s immune depletion.

In addition to augmenting the humoral immune response, MESC also increased phagocytic activity of neutrophils which suggests that it may enhance phagocytic activity of polymorphonuclear leukocytes and other phagocytic cells like macrophages. PMNs which can engulf, kill and digest microorganisms are usually the primary granulocytes attracted to invading pathogens by chemotaxins for destruction and elimination of the pathogens [35]. Phagocytic activity provides another line of defense against pathogenic invasion and contributes to strengthening the body’s immunity.

Besides the phagocytic actions of these cells at sites of inflammation or microbial invasion, the extent of their migration is equally important. Fortunately, the effect of MESC on leukocyte migration also showed it may enhance cell migration under inflammatory stimulus. Our results showed that treatment with MESC increased total and differential cell counts at the sites of injection of the inflammogen. The import of this action is that MESC may likely quicken cellular response to inflammation and microbial invasion in addition to stimulating and enhancing their immunological mechanisms such as phagocytosis. These actions clearly indicate immune-boosting properties of MESC.

The immunomodulatory activity of MESC alone was comparable to that of levamisole, a standard immunomodulatory agent. Levamisole, an immunostimulant, has the tendency to restore depressed immune function of the B-lymphocytes, T-lymphocytes, monocytes and macrophages [35]. The combined treatment of artesunate, a derivative of artemisinin with immunomodulatory effects [17], and MESC exhibited enhanced immunomodulatory activity on phagocytic activity of PMNs. The enhanced effect on phagocytic index elicited by the artesunate-MESC combination could be due to increased phagocytosis by the PMNs (neutrophils) exhibited by both agents. The PMNs being the first of the blood leucocytes to enter an inflamed cellular area can exhibit appreciable modulation of pro-inflammatory cytokines such as TNF-α, IL-1 and IL-10. Modulation of cytokines by artesunate could be a possible mechanism of its immunomodulatory actions since previous studies have reported the immunomodulatory effects of artesunate on cytokine production [36, 17]. Although the scope of this study may not permit elucidation of the specific mechanisms associated with the actions of MESC on humoral and cell-mediated immunity, augmentation or modulation of the immune system is supposedly mediated through opsonization, direct neutralization of antigen, agglutination of antigen and activation of complement system to cause lyses and death of antigenic cells [37]. Additionally, the leaf extract of *S. cayennensis* has been reported to possess antimalarial activity [19], it therefore, implies that the combined effect of MESC and artesunate may not only stimulate immune system, but also offer a good synergistic effect in the eradication of malaria parasites, thus proffering a possible new usage. In sub-Saharan Africa where there is high prevalence of malarial scourge, this combination will be a good therapeutic advantage especially to rural dwellers.

Studies on DTHR showed that MESC suppressed DTHR in treated animals. DTHR is a feature of chronic inflammation. In chronic inflammatory conditions such as rheumatoid arthritis, there is usually the manifestation of delayed hypersensitivity in the cells [38]. DTHR is a T-cell mediated event and the extent to which MESC or its constituents interferes with such reactions is not clear to us. The inhibitory and suppressive actions of MESC on DTHR induced by SRBCs could arise from mechanisms associated with its anti-inflammatory effect and may contribute to the usefulness of this plant in disorders of chronic inflammation such as arthritis and other musculoskeletal pains [18, 20]. Elsewhere, artesunate and other artemisinin derivatives have been shown to markedly suppress DTHR on chronic administration in laboratory animals [17].

The results of qualitative phytochemical analysis revealed the presence of carbohydrates, glycosides, flavonoids, saponins, alkaloids, terpenoids and steroids. Although no constituents could be linked to the immunomodulatory properties of the plant at this stage, studies have shown that different types of flavonoids may stimulate human peripheral blood leukocyte proliferation [39, 40]. Flavonoids have reported to cause increase in the helper T cells, interleukin 2 (IL-2), interferon and macrophages; hence they are useful in several diseases of immune dysfunction [41–43].

Acute toxicity studies of MESC showed an estimated LD_50_ greater than 5000 mg/kg, which is an indication of relative safety and remote risk of acute intoxication.

## Conclusion

In conclusion, this study showed that the leaves of *S. cayennensis* exhibited potent immunomodulatory properties. Combination of the extract with artesunate showed synergistic immunomodulatory effect, and could offer additional benefit in malaria treatment.
